# Pulmonary Arteriovenous Malformation in a Patient with Suspected Hereditary Hemorrhagic Telangiectasia: A Case Report

**DOI:** 10.21980/J8M353

**Published:** 2021-01-15

**Authors:** Nadia Zuabi, Sean Ryan Thompson, Alexa Lucas, Alisa Wray

**Affiliations:** * University of California, Irvine, Department of Emergency Medicine, Orange, CA

## Abstract

Hemoptysis is a common presenting symptom of hereditary hemorrhagic telangiectasia (HHT), an often overlooked diagnosis in the emergency setting. Patient history often includes telangiectasias, epistaxis, visceral lesions, and a family history of similar findings. Here, we review a case of HHT in a young woman, presenting initially with hemoptysis. Imaging was significant for large left upper lobe arteriovenous malformation (AVM), requiring patient admission and interventional radiology embolization of the AVM. The purpose of this report is to highlight a diagnosis that is important not to miss in the emergency department.

**Topics:**

Pulmonary arteriovenous malformation, hereditary hemorrhagic telangiectasia, Osler-Weber-Rendu syndrome.

**Figure:**
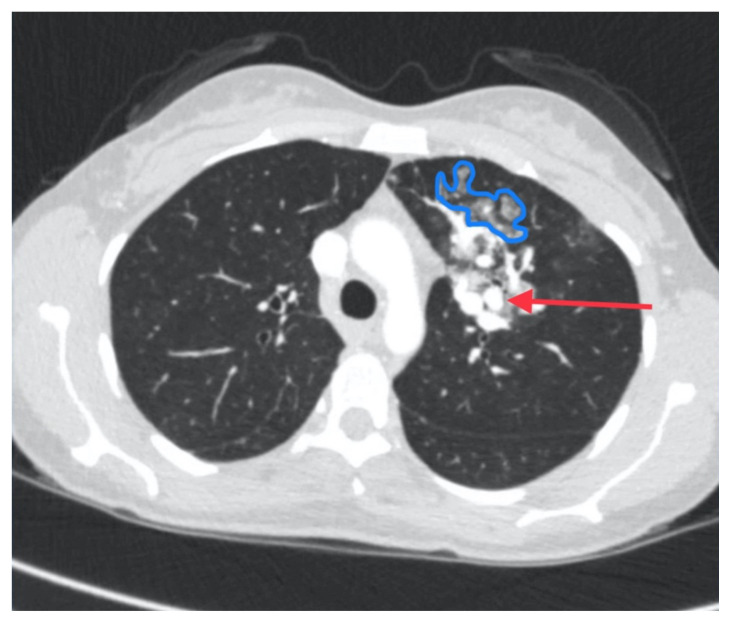
Annotated Video Link: https://youtu.be/qHBnBdoVkHwUnannotated Video Link: https://youtu.be/ol-x7Ulrpc4

## Brief introduction

While hemoptysis is often associated with infection (bronchitis, pneumonia, lung abscess), neoplasm (primary lung cancer, metastasis), and pulmonary embolism, it is frequently the presenting symptom in cases of HHT, an autosomal dominantly inherited condition characterized by a multitude of vascular malformations throughout the body.^1^ While the prevalence of this condition is between 1:5000 and 1:8000, this condition is often a missed diagnosis in the emergency department setting.^2^ A combination of a thorough history, the multi-system physical exam for skin, respiratory, neurological, and abdominal findings, and imaging may reveal a constellation of findings confirming the diagnosis. While many associated findings are evident on chest computed tomography, contrast-enhanced pulmonary angiography is the gold standard for diagnosis.^1^ In this report, we discuss the presentation of a patient with a history and imaging results that support the diagnosis of HHT in the emergency setting.

## Presenting concerns and clinical findings

We present a case of a 30-year-old female with a past medical history of asthma who presented with a two-day history of hemoptysis. The patient was seen the day before presentation at an outside hospital where she received a chest x-ray and was discharged home. She quantified coughing up approximately one-two cups of blood. Symptoms were associated with anxiety and shortness of breath. The patient also reported a personal and family history of frequent epistaxis. She denied any history of gastrointestinal bleeding. The patient denied any rashes but noted that she had telangiectasia on the right palm. The patient denied chest pain or fevers. She also denied a history of deep vein thrombosis or pulmonary embolus. She was not taking any blood thinners.

## Significant findings

Initial vital signs were unremarkable, including oxygen saturation of 98% on room air. The patient did not exhibit any signs of respiratory distress, and the lungs were clear to auscultation bilaterally. Labs were obtained, which showed normal hemoglobin at 15.8. Computed tomography (CT) of the chest (Video 1) showed a large left upper lobe arteriovenous malformation (AVM) with large feeding arteries and tortuous dilated draining veins (red arrow) measuring up to 3.8cm. Imaging also demonstrated nonspecific multifocal ground-glass opacities, which may have represented pulmonary hemorrhage (blue outline) from AVM without evidence of contrast extravasation to suggest active bleeding.

## Patient course

The patient was admitted to medicine service on the telemetry floor, and pulmonology and interventional radiology (IR) were consulted. IR performed a multi-vessel coil embolization of the AVM. During admission, the patient had episodes of desaturation to the high eighties on room air, which improved with two liters nasal cannula. She was weaned off oxygen upon discharge. Given the patient met Curaçao diagnostic criteria, pulmonology suspected hereditary hemorrhagic telangiectasia (HHT), also known as Osler-Weber-Rendu syndrome. She was referred for outpatient genetic testing upon discharge.

## Discussion

Hereditary hemorrhagic telangiectasia is an autosomal dominant disease that manifests as a variety of vascular defects in the body. The Curacao diagnostic criteria can be used to clinically diagnose affected patients and include epistaxis, telangiectasia, visceral lesions, and family history.^1^ Visceral lesions can appear in the gastrointestinal, pulmonary, hepatic, spinal, or cerebral organs. Prevalence of HHT is estimated to be between 1:5000 and 1:8000.^2^ One study by Mora-Lujan et al. studied gender differences regarding organ involvement. They found women had more severe hepatic involvement and more prevalence of pulmonary AVMs requiring procedural intervention.^3^

Arteriovenous malformations are a potential complication of HHT and can lead to adverse consequences depending on their origin. Pulmonary AVMs can lead to embolic stroke and brain abscess, given blood is passing through an anomalous track rather than the normal pulmonary circulation. Cerebral AVMs are a cause of hemorrhagic stroke, and hepatic AVMs have been identified as a cause of high-output cardiac failure.^2^ Although not seen in the case above, severe anemia can also be seen in patients with HHT. Of those patients with anemia, epistaxis was the most common cause. Treatment is supportive and includes pressure, packing, and cauterization.^4^

Pulmonary AVMs, specifically, can be identified on multiple imaging modalities, including chest radiography, computed tomography, contrast-enhanced pulmonary angiography (CTA), and magnetic resonance angiography.^5^ This patient was discharged from an outside hospital after receiving an x-ray and presenting with similar symptoms. Although it is not known what that chest x-ray showed, it is prudent for the emergency physician to keep HHT under the differential diagnosis for patients presenting with hemoptysis. Knowledge of this outside imaging could have potentially strengthened our approach to this case. This is especially true given pulmonary AVMs are known to be under-reported despite being observed in 15–45% of patients with HHT.^6^ Of note, not all pulmonary AVMs require treatment. Treatment is usually determined based on the diameter of the arteries feeding into the AVM. When these arteries are over 2–3 mm in diameter, embolization by IR is typically preferred to observation.^7^ Surgical options are also pursued for failed embolization, life-threatening circumstances, or when IR is not available. Surgical intervention is also preferred in specific patient populations that should avoid contrast CT such as pregnant patients.^8^

## Supplementary Information










