# Supplementary figures and images for: Pulmonary Arteriovenous Malformation in a Patient with Suspected Hereditary Hemorrhagic Telangiectasia: A Case Report
Source: J Educ Teach Emerg Med. 2021 Jan 15;6(1):V9–V11. doi: 10.21980/J8M353 (PMC10332757; doi:10.21980/J8M353)

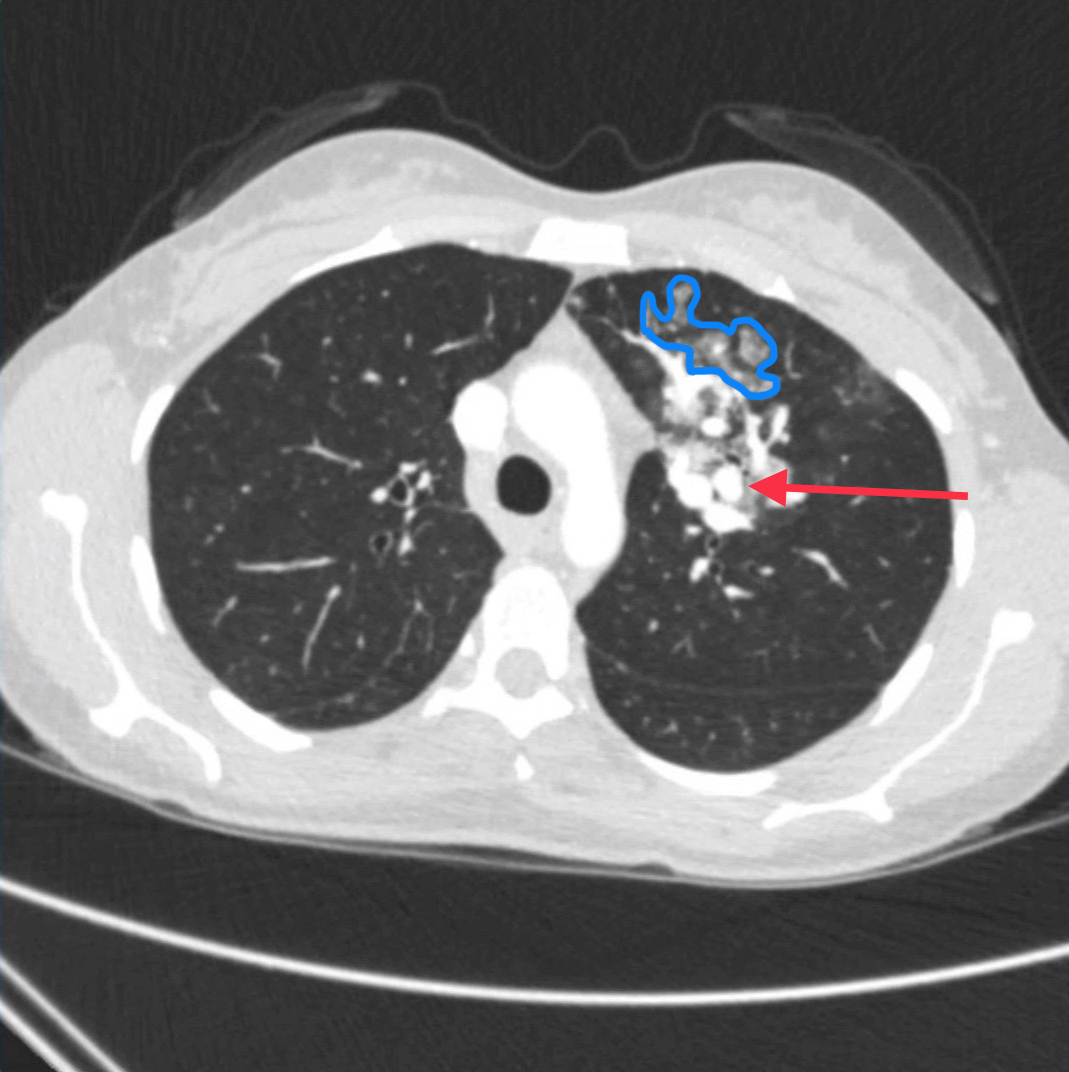

Supplement: Supplementary file 1 [file jetem-6-1-v9-supp1.jpeg]

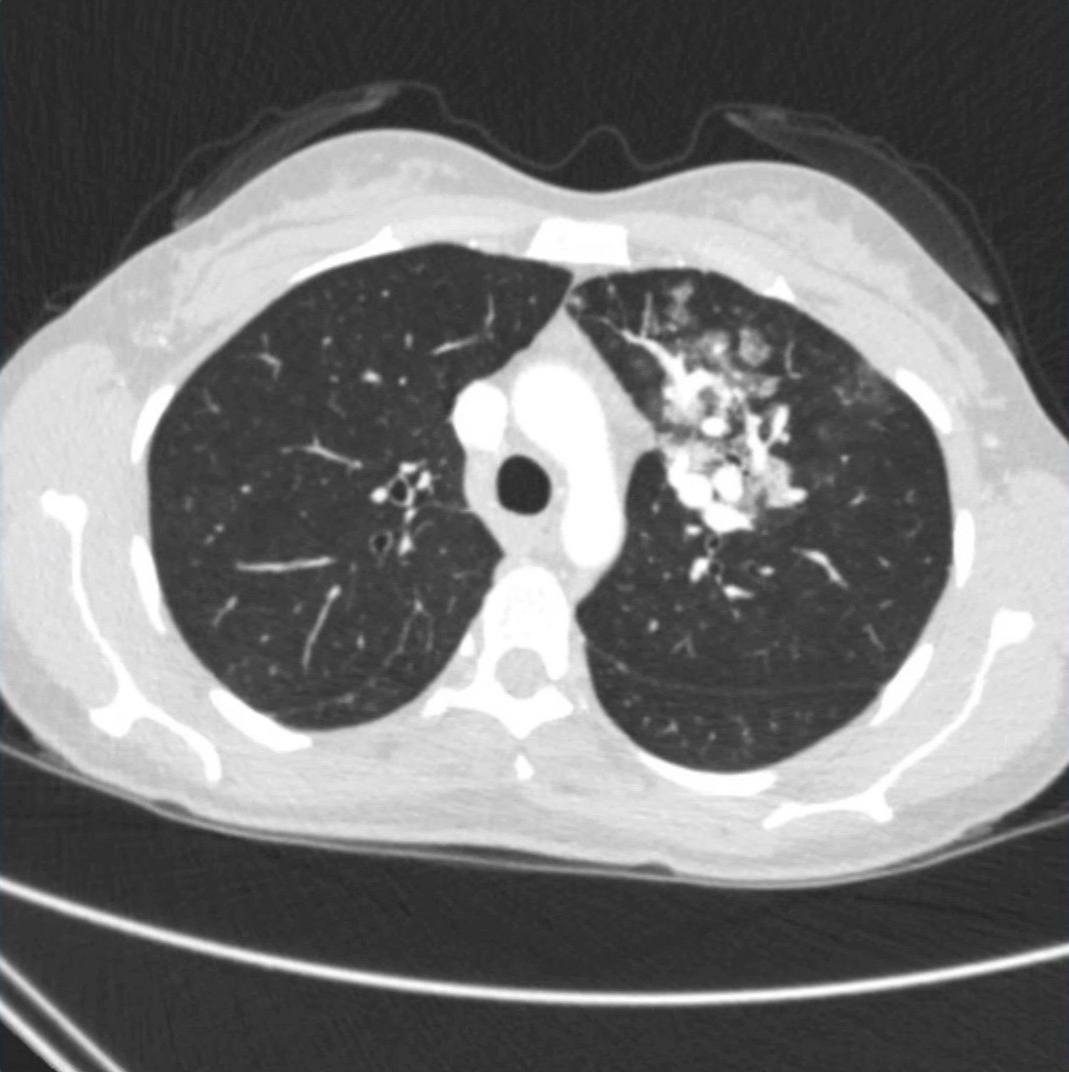

Supplement: Supplementary file 4 [file jetem-6-1-v9-supp4.jpeg]
